# A mixed-method approach to assess factors associated with food provision to children during weaning in Singapore

**DOI:** 10.1007/s00394-026-03927-7

**Published:** 2026-03-02

**Authors:** Angel Lin Fung Chi, Allan Lim, Sharron A. Kuznesof, Chris J. Seal, Iain A. Brownlee

**Affiliations:** 1 Nestlé Singapore, 15A Changi Business Park Central 1, #05-02/03 Eightrium @ Changi Business Park,, Singapore, 486035 Singapore; 2 Newcastle Research and Innovation Institute, Devan Nair Building, Singapore, 600201 Singapore; 3 Applied Social Sciences - School of Natural and Environmental Sciences, Newcastle University, Newcastle upon Tyne, NE1 7RU UK; 4 Human Nutrition and Exercise Research Centre, Population Health Sciences Institute, Newcastle University, Newcastle upon Tyne, NE2 4HH UK; 5 Faculty of Science and Environment, Northumbria University, Newcastle-upon-Tyne, NE1 8ST UK; 6 Faculty of Health and Life Sciences, Northumbria University, Newcastle-upon-Tyne, NE1 8ST UK

**Keywords:** Diet quality, Dietary intake, Complementary feeding, Weaning, Food-based guidelines, Nutrient-based guidelines

## Abstract

**Purpose:**

The complementary feeding period is a key transitional phase in which lifelong dietary preferences are developed which shapes disease risk in later life. This study aimed to use cross-sectional data to examine how maternal characteristics are associated with the diet quality of their offspring.

**Methods:**

Analysis of cross-sectional data in Singaporean mother-child dyads was conducted, alongside individual in-depth interviews to explore mothers’ perceptions and beliefs on food provisioning. Data from 488 Singaporean mother-child dyads (aged 6-24mo), were collected. Food intake (assessed by food frequency questionnaires), anthropometric and questionnaire data were collected during on-site visits. In-depth interviews were carried out in a sub-group of mothers (*n* = 12) to better understand dietary habits, choices, motives and influences related to their food provisioning. Pearson/Spearman correlation coefficients were used to explore diet quality relationships in the mother-child dyads. Multiple linear regression models were applied to examine the predictors for the diet quality of a child.

**Results:**

Diet quality of children was significantly affected by maternal age, education, BMI, breastfeeding practice, and household income. The total diet quality scores in mother-child dyads were correlated (*r* = 0.176, *p* < 0.001), contributed primarily by whole grains and fruit (*r* = 0.330 and *r* = 0.325 respectively, *p* < 0.001), and vegetable (*r* = 0.125, *p* = 0.006). Breastfeeding was the strongest predictor of a child’s diet quality (β = 0.195; *p* < 0.001), and mothers valued breastfeeding qualitatively as a means to improve the bonding and well-being of their children.

**Conclusion:**

Multiple factors influence diet quality in children during complementary feeding, including breastfeeding, maternal age, BMI status, dietary habit and educational attainment.

**Supplementary Information:**

The online version contains supplementary material available at 10.1007/s00394-026-03927-7.

## Introduction

Parents and caregivers are the gatekeepers of food provisioning for their children [1–3]. While maternal habit appears to be a major driver of that of children, it is currently unclear when maternal dietary habits may start to have an influence. Some observational studies reveal that the diet patterns of infants and toddlers are associated with the maternal dietary habit [4–7] suggesting that the early-life feeding period is a critical window. North & Emmett (2000) suggested that diet quality among young children may be linked with social conditions in which they live [8]. Others have proposed that the genetic makeup [9], BMI status^,^ [10–12], socio-psychological well-being of mother [13, 14], education levels [15–17], feeding strategy [18], food availability at home [19, 20] and other aspects of the shared food environment all contribute to the diet quality of a child. Characteristics such as mothers’ weight status [21] and perceptions of positive food choices [22] may influence their own food choices, and thus, their overall diet quality which in turn potentially shapes the dietary pattern of their growing children. To date, a large proportion of current literature on food choice and belief focuses predominantly on Western cultures and the findings cannot be generalized to the rest of the world, including Southeast Asia [23].

Diet quality estimates could provide a better representation of the overall “idealness” of dietary intake [24] regarding adherence to food- or nutrient-based guidelines. The GUSTO Cohort Study [15], examining 561 toddlers at 18 months in Singapore, showed that maternal education, household income, maternal pre-pregnancy BMI, and ethnic origin affected the quality of a child’s diet. However, such relationships between mothers and their weaning children (at the age of 6 to 24 months) are yet to be determined. Our previous, cross-sectional work [25] reported the overall adherence to food- and nutrient-based based guidelines (“diet quality”) of mothers and their children but has not considered the major maternal qualitative and quantitative characteristics associated with this in-depth.

The position of mothers as authority figures and their impact on model behaviours appear to be important correlates of dietary habit in older children [4, 26, 27]. The impact of the own behaviours and associated factors (including health beliefs and socio-economic measures) could be particularly impactful during complementary feeding [28–30], as this represents a period of time when infants have not yet developed their own food choice preferences [31]. Exploring maternal decision-making around complementary food provision requires both qualitative and quantitative information [32, 33]. As such, the purpose of this work was to consider how diet quality in mothers, alongside socio-economic metrics and health beliefs, were associated with the appropriateness of food provision to infants during complementary feeding using a mixed-method approach.

The specific aims of this study were: (a) to determine the association between maternal demographic, health and socio-economic characteristics (e.g., age, BMI, education attainment) and child diet quality ; (b) to determine the relationship between the diet quality of children and that of their mothers, including sub-components of adherence to national dietary guidelines and (c) to qualitatively explore the perceptions and beliefs of mothers on their choice of food for their children. This adds to our primary analysis by considering a range of quantitative, categorical and qualitative factors that are associated with infant diet quality during complementary feeding and helps to further explore the complex relationship between maternal and child dietary habits in an under-researched population.

## Methods

### Participants and data collection

#### Quantitative data

Full details of the participant recruitment and the experimental approach have been presented elsewhere [25]. Ethical approval of this project was sought in June 2017 by the School of Agriculture, Food and Rural Development Research Ethics Committee, Newcastle University [Ref: 17-LIN-037], in alignment with the national ethical framework requirements [34]. The participants provided written informed consent before the start of the study. A total of 484 mother-child pairs were recruited. Mothers of children (aged 6–24 months) completed the food frequency questionnaires and provided socio-demographic, other background information, and dietary intake data during the induction visits [25]. All mothers who participated in the study were made aware of the follow up through interviews.

## Qualitative data

**Fig. 1:**
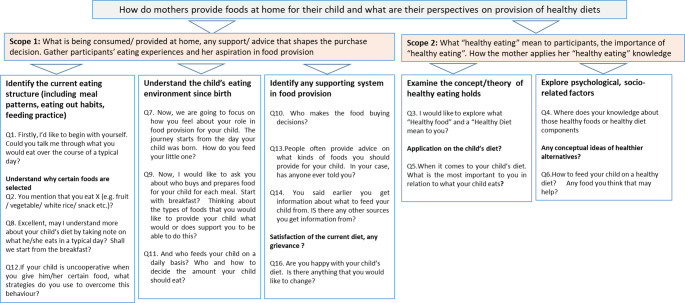
Qualitative data collection using individual in-depth interviews

A final sample of, twelve agreed to be involved in the ‘individual in-depth interview’(IIDIs) with a purposeful approach undertaken to include equal numbers of mothers in older and younger age groups. IIDIs allow detailed interaction between interviewer and interviewee [35]. IIDIs used open-ended questions to explore (i) the child’s current eating structure, including their daily food intake, (ii) the relationship between mother and child’s diet and (iii) maternal attitudes and perceptions about healthy eating, its application in the child’s diet and understanding of nutritional knowledge, as well as the feeding practice (see Fig. 1). Further questions discussed choices, motives and influences of mothers’ decision-making process and the sources of support in food provisioning at home. No repeat interviews were carried out. Analysis of dietary intake data represents secondary analysis of findings from our previous study [25], while exploration of qualitative IIDI data has not yet been reported. All interviews were carried out by one, trained female researcher (ALFC), who participants had been introduced to already through the consenting process and their prior involvement in the quantitative data elements of this work. Participants were all aware of the interviewer’s existing job role and the rationale for the research. The interviews primarily took place in the research facility but were otherwise organised in quiet, private settings due to participant availability. Participants were chosen based on their prior interest noted at the quantitative data collection visits and were purposively sampled based on a mothers’ education, employment status, household income and grocery spending to give a balanced distribution across the study population. Of the studied cohort, it was found that 210 mothers with a child aged 6 to 24 months at the time of organising interviews. Of these 210, twenty -three mothers were successfully contacted by telephone with positive responses, of which 14 accepted to participate. A further written consent was given before proceeding to the interview. Two were not able to meet for interviews. As such, there were 12, face-to-face interviews conducted, with 6 in each sub-group. IIDIss took c.60 minutes. Figure 1 shows the data collection using IIDIs.

## Sample size

The sample size calculation in evaluating the diet in mother-child dyads was presented previously [25] and aimed for a nationally representative sample by n-value. For individual in-depth interviews (IIDIs), theoretical saturation has previously been reported within the first six interviews [36]. In this cohort, twelve interviews were conducted to capture the variations between the participants [37] prior to analysis in order to limit the chances of saturation not being met. This number (*n* = 12) has also been suggested to mitigate maximum variation in qualitative cohorts [37].

Date Processing - Quantitate Data.

Diet quality scores in mother-child dyads were calculated using the healthy eating index (HEI- SG) as described previously [25], with original dietary data collection from food frequency questionnaires [38, 39]. Briefly, energy-adjusted food and nutrient intake were compared to national, age- and sex-specific nutrition recommendations [40–42], with continuous scores of adherence to guidelines for each of ten components based on adherence (full adherence = 10.0 points; adherence did defined threshold – 0.0 points) calculated. An overall score was calculated for each participant based on the total of the component scores, providing a final score out of a possible 100.0. A higher adequacy component score in the index construct represents better adherence to the national food-based guidelines for mothers and infants [40–42]. This approach to diet quality scoring has not been externally validated but the questionnaires have previously been used and validated for studies in related populations [39, 43].

Primary processing of intake data from food frequency questionnaires (FFQs), collected at a single time point in-person from mothers at study visits, was carried out by the lead researcher (ALFC), with agreement of representative compositional data and food group conversion from the lead supervisor (IAB). Categorical data such as maternal sociodemographic characteristics and breastfeeding practice were coded numerically to support statistical analysis due to software requirements for data input.

## Data processing–qualitative data

IIDIs were considered with a focus on consistent questioning for data collection to inform subsequent content analysis. Maternal socio-psychological data such as mothers’ well-being, beliefs and perceptions in home food provisioning were obtained in IIDIs. The interview schedule was developed based on similar previous work [44].

The audio-recordings/field (without further participant comment) notes from each interview were transcribed and examined thematically using the qualitative data management package NVivo 12 (QSR International Pty. Ltd, Melbourne, Australia). Data were compared across each stage of the analytic process (i.e., nodes, quotes, codes, categories, themes) to produce abstract concepts. The lead researcher (ALFC) interpreted of the unstructured data from the transcripts by identifying the key words, looking into common quotes, non-verbal notes as well as commonly used words to systematically develop nodes within NVivo. Coding of these nodes involved the desegregation of textual data in which conceptually similar data were connected in the respective nodes. There could be several nodes within the same category. In order to generate the themes, the lead researcher shaped and examined the nodes. Scrutiny of the texts continued until no more new ideas were generated from the dataset. Oversight of this process was provided by a supervisor with extensive experience in qualitative data handling and NVivo usage (SAK).

### Statistical analysis

Statistical analysis was performed using the Statistical Package for Social Science (SPSS) version 25.0 for Windows and statistical significance for all the tests was defined at a p-value of < 0.05. ANOVA (analysis of variance) tests were performed on child’s diet quality scores with Kruskal-Wallis tests to determine if there were statistically significant differences between subgroups on a series of categorical maternal characteristics (e.g., demographic, anthropometric, and habitual variables). Following testing for normality through use of Kolmogorov–Smirnov test, both Pearson and Spearman correlation coefficients were used to explore dietary intake and diet quality relationships in mother-child dyads. Global and contextual outliers were excluded following confirmation with original data collection entries.

Correlations were considered weak if *r* < 0.30, moderate if ≥ 0.3–0.49 and strong if ≥ 0.5) [45]. A multivariate linear regression model assessing the independent variables (e.g., maternal BMI, age, ethnicity, education attainment, employment status, household incomes, breastfeeding practice, dwelling and purchase practices) to determine the predictors of the diet quality of a child with covariates including a child’s sex, age, and BMI-z score were adjusted. Stepwise regressions were used due to their applicability to analyse multiple, putatively-dependent variables. The potential confounders of socio-demographic and biological variables were controlled in the multiple linear models. and variables with *p*-value < 0.05 were entered into the final model to assess the independent effects of each predictor on the diet quality scores of a child following the removal of outliers. Unstandardized and standard β coefficients were reported and a p-value < 0.05 (two-tailed) was considered significant.

## Results

### Demographic and dietary data

Of the 484 child-mother pairs, most (87.6%) were ethnically Chinese, followed by Indian (6.6%) and Malay (3.9%). Over half (55.2%) of the mothers cohort were degree holders, with a household income between S$9001 to S$20,000. Information about the socio-demographic, habitual and diet-related behaviours is shown in Table 1.

**Table 1:** Maternal characteristics data including socio-demographic, habitual and diet-related behaviours in both quantitative and qualitative analyses

Variables		Quantitative (*n* = 484)		Qualitative (*n* = 12)	
	Category	Proportion (*n*-values)	Percentage	Proportion (*n*-values)	Percentage
Ethnicity	Chinese: Malay: Indian: Others	424: 32: 19: 9	87.6: 6.6: 3.9: 1.9^2^	12: 0: 0: 0: 0	100.0: 0.0: 0.0: 0.0
Marital status	Married: Divorce	483: 1	99.8: 0.2	12: 0	100.0: 0.0: 0.0: 0.0
Education	Basic: Intermediate: Advanced I: Advanced II^1^	80: 80: 267: 57	1.5: 16.5: 55.2: 11.8	0: 0: 8: 4	0.0: 0.0: 66.7: 33.3
Employment status	Others: Home maker: Part time: Full time^2^	9: 96: 37: 342	1.9: 19.8: 7.6: 70.7	0: 3: 0: 9	0.0: 25.0: 0.0: 75.0
Household income	Level I: Level II: Level III: Level IV^3^	29: 217: 183: 53	6.0: 45.0: 38.0: 11.0	0: 4: 8: 0	0: 33.3: 66.7: 0
Type of residence	House: Private condominium: HDB^4^	27: 53: 404	5.6: 11.0: 83.5	0: 3: 9	0.0: 25.0: 75.0
Weekly spending on food grocery/head	Low: Medium: High: Very High^5^	121: 218: 86: 59	25.0: 45.0: 17.8: 12.2	3: 9: 0: 0	25.0: 75.0: 0.0: 0.0
Meal preparation/day	No: Sometime: Yes	58: 108: 318	12.0: 22.3: 65.7	1: 4: 7	8.3: 33.3: 58.3
Purchase of ‘healthier choice’ produce	Never: Sometimes: Very Often	91: 192: 201	18.8: 39.7: 41.5	2: 7: 3	16.7: 58.3: 25.0
Purchase of ‘organic’ produce	Never: Sometimes: Very Often	164: 207: 113	33.9: 42.8: 23.3	1: 6: 5	8.3: 50.0: 41.7
Understanding of dietary guidelines	None: Limited: Basic: Advanced	70: 135: 256: 23	14.5: 27.9: 52.9: 4.8	1: 2: 9: 0	8.3: 16.7: 75.0: 0.0
Breastfeeding practice	First 6 months: ever breastfed: never breastfed	336: 136: 12	69.4: 28.1: 2.5	8: 4: 0	66/7: 33.3: 0.0
Health status (self-reported)	Bad: Not Good: Good: Very Good	1: 60: 387: 36	0.0: 12.4: 80.0: 7.4	0: 0: 9: 3	0.0: 0.0: 75.0: 25.0
Satisfied with current weight	Not satisfied: Slightly: Satisfied: Very much	198: 138: 120: 28	40.9: 28.5: 24.8: 5.8	6: 2: 3: 1	50.0: 16.7: 25.0: 8.3
Self-reported diet rating	Poor: Fair: Good: Very Good	7: 221: 220:36	1.4: 45.7: 45.4: 7.4	0: 7: 5: 0	0.0: 58.3: 41.7: 0.0
Self-induced restrictive diet	Yes: No	61: 423	12.6: 87.4	6:6	50.0: 50.0
Exercise	Light: Moderate: Vigorous	429: 44: 11	88.6: 9.1: 2.3	12: 0: 0	100.0: 0.0: 0.0
Smoker	Yes: No	14: 470	2.9: 97.1	0:12	0.0: 100.0
Drinker	Yes: No	6: 478	1.2: 98.8	0: 12	0.0: 100.00

^1^Basic: schooler leavers; Intermediate: polytechnic or diploma holders; Advanced I: degree holders; Advanced II: postgraduate degree holders. ^2^Full time: ≥ 44 hours/week; Part time: < 44 hours/week. ^3^Household income Level I: <$ 3500pcm; II: $3501- ≤ $9000pcm; III: $9001- ≤ $15000pcm; IV: $15001- ≥ $20001pcm. ^4^HDB: public housing developed by Housing Development Board of Singapore. ^5^Low: < $100; Medium: $101- 200; High: $201–350; Very High: $351–500

### Associations between maternal characteristics and the quality of child’s diet

Table 2 shows that children of older mothers had higher overall total diet quality scores (HEI-SG) than those children of younger mothers (*p* = 0.026), with a median score of (65.0 (63.8–66.2)) and (62.1 (59.9–64.4)) respectively. Children of mothers who were of Chinese ethnic origin scored a statistically higher HEI-SG-C total median score (65.0 (63.9–66.1)) when compared with non-Chinese (59.8 (56.4–63.2), *p* = 0.001), with statistically higher component scores for sodium (*p* < 0.001) and added sugar (*p* < 0.001). Children of highly educated mothers had statistically higher median HEI- SG-C total score (66.8 (63.4–70.1), *p* = 0.009), with significantly lower component scores for sodium (*p* = 0.036) and added sugar (< 0.001) but higher score for fruit (< 0.001). The pairwise comparisons confirmed that children of mothers who received basic education scored significantly lower for added sugar and fruit than mothers of advanced II education (*p* < 0.002) (see Supplementary Table A).

**Table 2:** Evaluation of child healthy eating index score (HEI-SG-C) by maternal demographic, socio-economic characteristic characteristics

Component	Age range			Ethnicity			Education^1^					Household Income				
	18-29y (*n* = 107)	30-50y (*n* = 377)		Non-Chinese (*n* = 60)	Chinese (*n* = 424)		Basic (*n* = 80)	Int.(*n* = 80)	Adv. I^3^ (*n* = 267)	Adv. II^4^ (*n* = 57)		Level I ^5^ (*n* = 29)	Level II^6^ (*n* = 217)	Level III (*n* = 183)	Level IV (*n* = 53)	
	Median IQR		p	Median IQR		p	Median IQR				p	Median IQR				p
Rice & alternatives	82.7 (58.4–100.0)	80.6 (60.2–100.0)	0.967	88.4 (65.5–100.0)	80.3 (59.1–100.0)	0.238	78.7 (58.7–100.0)	77.9 (57.9–100)	82.7 (63.1–100.0)	75.9 (54.8–100.0)	0.430	83.5 (65.1–100.0)	81.4 (61.7–100.0)	80.9 (60.8–100.0)	74.3 (52.3–97.4)	0.420
Whole grains	4.3 (0.0–44.0)	8.0 (0.0-55.3)	0.271	1.0 (0.0-32.7)	7.9 (0.0-55.6)	0.074	0.0 (0.0-32.7)	7.2 (0.0-63.4)	7.7 (0.0-55.8)	17.2 (0.0-53.2)	0.155	11.2 (0.0-31.6)	2.0 (0.0-47.6)	12.8 (0.0-83.8)	22.2 (0-53.2)	0.053
Meat & Alternatives	88.8 (46.1–100.0)	89.5 (53.7–100.0)	0.660	75.8 (40.4–100.0)	90.3 (53.8–100.0)	0.071	89.0 (60.9–100.0)	76.5 (37.6–100.0)	91.2 (56.7–100.0)	93.0 (40.8–100.0)	0.130	74.3 (57.3–100)	88.5 (47.2–100.0)	90.9 (55.9–100)	89.3 (59.8–100)	0.600
Dairy & Alternatives	72.5 (60.3–87.4)	70.1 (58.6–82.7)	0.205	69.1 (55.9–79.1)	71.2 (58.7–84.2)	0.347	70.9 (58.5–82)	72.1 (60.2–84.1)	71.2 (58.9–84.9)	68.3 (53.9–78.3)	0.412	66.8 (55–78.0)	71.8 (58.6–86.9)	69.8 (59-81.8)	71.2 (62.1–79.6)	0.453
Vegetables	82.8 (51.7–100.0)	93.8 (53.1–100.0)	0.32	78.5 (31.5–100.0)	91.5 (54.5–100.0)	0.218	74.1 (41.2–100.0)	90.4 (47.4–100)	88.2 (56.0-100.0)	100.0 (69.3–100.0)	0.053	58.7 (26.2–100)^a^	80.1 (51.9–100.0)^ab^	99.7 (59.1–100.0)^b^	100.0 (63.6–100.0)^b^	0.009
Fruit	51.5 (22.2–83)^a^	67.8 (35.9–100.0)^b^	0.001	77.2 (29.5–100.0)	61.1 (33.4–100.0)	0.309	58.5 (21.2–100.0)^a^	51.8 (28-96.4)^a^	60.5 (35.7–96.8)^a^	93.5 (62.3–100.0)^b^	< 0.001	61.2 (29.4–100)	62.4 (26.3–100.0)	60.9 (38.2–100)	78.6 (37.0-100)	0.274
Sodium	100.0 (0.0-100.0)	100.0 (100.0-100.0)	0.546	100 (0.0-100.0)^a^	100.0 (100.0-100.0)^b^	< 0.001	100.0 (0.0-100.0)	100.0 (0.0-100.0)	100.0 (100.0-100.0)	100.0 (100.0-100.0)	0.036	0.0 (0.0-100.0)^a^	100.0 (100.0-100.0)^b^	100.0 (100.0-100.0)^b^	100.0 (100.0-100.0)^b^	0.003
Total Fat	100.0 (0.0-100.0)	100.0 (0.0-100.0)	0.472	100.0 (0.0-100.0)	100.0 (0.0-100.0)	0.704	100.0 (0.0-100.0)	100.0 (20.8–100.0)	100.0 (0.0-100.0)	100.0 (0.0-100.0)	0.949	100.0 (41.2–100.0)	100.0 (0.0-100.0)	100.0 (25.5–100.0)	100.0 (32.1–100.0)	0.199
Saturated Fat	0.0 (0.0-28.4)	0.0 (0.0-39.4)	0.193	0.0 (0.0-51.5)	0.0 (0.0-37.3)	0.314	0.0 (0.0-47.6)	0.0 (0.0-41.5)	0.0 (0.0-32.7)	0.0 (0.0–50.0)	0.651	0.0 (0.0-5.7)	0.0 (0.0-44.7)	0.0 (0.0-32.7)	0.0 (0.0-39.4)	0.422
Added Sugar	97.1 (93.3–99)	97.2 (92.7–99.2)	0.913	94.2 (82.0-97.3) ^a^	97.5 (93.5–99.3) ^b^	< 0.001	94.3 (85.4–97.9)^a^	96.7 (91.1–98.9)^ab^	97.6 (93.9–99.3)^b^	98.2 (93.3–99.7) ^b^	< 0.001	89.9 (83.2–97.0)^a^	96.5 (92.9–98.9)^b^	98.0 (94.2–99.3)^b^	97.8 (93.4–99.6)^b^	< 0.001
Overall (Total)*	62.1 (59.9–64.4)	65.0 (63.8–66.2)	0.026	59.8 (56.4–63.2)	65.0 (63.9–66.1)	0.001	61.2 (58.5–63.9)^a^	62.7 (59.9–65.4) ^ab^	65.3 (64.0-66.6)^b^	66.8 (63.4–70.1)^b^	0.009	57.7 (53.7–61.8)^c d^	62.9 (61.2–64.6)^c^	66.6 (65.1–68.1)^a b^	66.4 (63.1–69.8)^a^	< 0.001

*ANOVA test, with mean and 95% confidence presented. All other data were compared with Kruskal-Wallis tests. Sub-groups with different superscripts (a,b) for education attainment and household income in a single row are statistically different (p < 0.05) by Mann-Whitney tests with Bonferroni’s post hoc test.^1^Basic: school leavers; Intermediate (Int.): polytechnic or diploma holders; Advanced I (Adv. I): degree holders; Advanced II (Adv. II): postgraduate degree holders. ^2^Level I <$ 3500pcm; Level II $3501- ≤ $9000pcm; Level III $9001- ≤ $15000pcm; Level IV $15001- ≥ $20001pcm

The HEI-SG-C total score was significantly (*p* < 0.001) higher in children of higher-income households (66.4 (63.1–69.8), primarily contributed by vegetable (*p* = 0.009), sodium (*p* = 0.003) and added sugar (*p* < 0.001) components. Sub-analyses on the household income against the HEI-SG-C confirmed that children of lowest-income households (Level I) consistently scored low for vegetables (i.e., Level III (*p* = 0.024) and Level IV (*p* = 0.045), sodium (i.e., Level II: *p* = 0.008; Level III: *p* = 0.001; Level IV: *p* = 0.020) and added sugar (i.e., Level II: *p* = 0.010; Level III: * P*  = < 0.001; Level IV: * P*= < 0.001) after Bonferroni adjustment (see Supplementary Table B). Table 3 shows that children of obese mothers had lower HEI-SG-C total median score (61.4 (58.9–63.8)) compared with children of mothers whose BMI was within the normal category (66.2 (64.7–67.7), *p* = 0.008), although no individual component score was statistically different across the BMI category.

**Table 3:** Child healthy eating index score (HEI-SG-C) by the maternal characteristics

	BMI status						Breastfeeding practice				Purchase habit of organic foods		
Component		Underweight^1^ (*n* = 41)	Normal^2^ (*n* = 236)	Overweight^3^ (*n* = 136)	Obese^4^ (*n* = 64)		First 6 months^5^ (*n* = 336)	Ever breastfeeding^6^ (*n* = 136)	Never^7^ (*n* = 12)		No (*n* = 164)	Yes (*n* = 320)	
		Median IQR				* P*	Median IQR			* P*	Median IQR		* P*
Rice & alt		84.3 (59.9–98.5)	83.7 (61.1–100.0)	80.5 (59.4–100.0)	71.9 (55.5–97.9)	0.468	79.7 (59.5–100.0)	84.1 (62.5–100.0)	93.1 (82.3–100.0)	0.235	80.8 (64.0–100.0)	80.6 (58.9–100.0)	0.724
Whole grains		26.0 (0.0-53.2)	9.0 (0.0-65.4)	3.7 (0.0–41.2)	4.5 (0.0–33.2)	0.157	8.5 (0.0–53.2)	4.2 (0.0–47.8)	3.1 (0.0–76.1)	0.660	0.0 (0.0–40.7)	11.7 (0.0–56.2)	0.004
Meat & alternatives		78.4 (59.9–100.0)	90.6 (50.6–100.0)	81.0 (52.7–100.0)	91.0 (57.1–100.0)	0.565	89.7 (51.2–100.0)	88.8 (56.7–100.0)	71.5 (57.6–89.7)	0.576	89.2 (55.4–100.0)	89.4 (51.6–100.0)	0.907
Dairy & alternatives		69.8 (61.7–80.4)	71.4 (58.8–83.4)	71.3 (58.4–83.8)	69.9 (58.5–85.3)	0.969	72.7 (60.1–86.8)^a^	68.3 (55.1–77.0)^b^	62.6 (58.8–72.9)^ab^	0.004	72.2 (60.8–83.3)	69.7 (58.2–84.0)	0.460
Vegetables		68.6 (48.6–100.0)	95.0 (59.2–100.0)	81.9 (48.7–100.0)	74.6 (46.5–100.0)	0.367	89.6 (53.7–100.0)	95.8 (51.6–100.0)	65.5 (47.7–85.9)	0.304	70.3 (45.2–100.0)	99.9 (59.8–100.0)	< 0.001
Fruit		51.2 (37.5–86.4)	69.8 (34.6–100.0)	60.1 (28.1–94.3)	63.8 (40.9–88.7)	0.283	68.7 (35.4–100.0)	56.1 (29.1–90.8)	43.0 (26.2–78.1)	0.051	58.5 (29.4–94.0)	65.2 (36.1–100.0)	0.150
Sodium		100.0 (100.0–100.0)	100.0 (100.0-100.0)	100.0 (100.0-100.0)	100.0 (0.0–100.0)	0.058	100.0 (100.0–100.0)^a^	100.0 (0.0–100.0)^b^	100.0 (0.0–100.0)^ab^	0.008	100.0 (0.0–100.0)	100.0 (100.0–100.0)	0.132
Total fat		100.0 (0.0–100.0)	100.0 (0.0–100.0)	100.0 (0.0–100.0)	100.0 (0.0–100.0)	0.771	100.0 (20.8–100.0)	100.0 (0.0–100.0)	0.0 (0.0–100.0)	0.030	100.0 (7.9–100.0)	100.0 (0.0–100.0)	0.109
Saturated fat		0.0 (0.0–28.4)	0.0 (0.0–47.2)	0.0 (0.0–37.3)	0.0 (0.0–10.7)	0.109	4.5 (0.0–52.4)^a^	0.0 (0.0–0.0)^b^	0.0 (0.0–0.0)^b^	< 0.001	0.0 (0.0-36.9)	0.0 (0.0–39.2)	0.461
Added sugar		98.0 (92.0–99.6)	97.4 (93.9–99.2)	96.7 (92–98.7)	97.5 (91.6–99.4)	0.161	97.3 (93.2–99.3)	97.1 (91.4–98.8)	97.3 (95.2–99.2)	0.475	95.7 (90.5–98.6)	97.8 (94.1–99.3)	< 0.001
Overall (Total)*		64.8 (61.7–67.9)	66.2 (64.7–67.7)^a^	62.9 (60.8–65.1)	61.4 (58.9–63.8)^b^	0.008	66.1 (64.9–67.4)^a^	60.6 (58.8–62.4)^b^	57.5 (50.3–64.7)^b^	< 0.001	62.8 (61-64.5)	65.2 (63.9–66.5)	0.033

*ANOVA test, with mean and 95% confidence interval. All other data were compared with Kruskal-Wallis tests. Superscripts (a, b) for BMI statuses and breastfeeding practices are statistically different (*p* < 0.05) by Mann-Whitney tests with Bonferroni’s post hoc test. ^1^Underweight refers to BMI below 18.5 kg/m2. ^2^Normal weight refers to BMI 18.5–22.9 kg/m^2^. ^3^Overweight refers to BMI between 23 and 29.9 kg/m^2^. ^4^Obese refers to BMI > 30.0 kg/m^2^. ^5^First 6 months: children who were breastfed for the first 6 months. ^6^Ever breastfeeding: children who were breastfed for less than 6 months. ^7^Never: children who were never breastfed

Children who were breastfed for the first 6 months had a significantly (*p* < 0.001) higher HEI-SG-C total score. Within the breastfed children group, a pairwise comparison showed that the ‘first 6-month’ group scored significantly higher for dairy and alternatives (*p* = 0.016), sodium (*p* = 0.007) and saturated fat (*p* = < 0.001) than those in the ‘ever’ breastfed group (see Supplementary Table C). Children whose mothers had a habit of purchasing organic foods scored significantly (*p* = 0.033) higher in total HEI-SG-C (65.2 (63.9–66.5) than those with mothers who never bought organic foods (62.8 (61-64.5). Lower child diet quality scores were noted with reduced component scores for whole grains (*p* = 0.004), vegetables (*p* < 0.001) and added sugar (*p* < 0.001). No significant correlation of child diet quality score was noted with differing maternal characteristics across factors like employment status, dwelling type, weekly grocery spending, meal preparation practices, knowledge of dietary guidelines and ‘healthier choice -purchase’.

### Correlation and regression analyses

Table 4 shows the component scores for whole grains and fruit in children were positively but weakly associated with the mothers’ HEI-SG-M total score (*r* = 0.285, *r* = 0.209, *p* < 0.001, respectively). The total diet quality scores in mother-child dyads were correlated (*r* = 0.176, *p* < 0.001), with ‘weak-to-moderate’ relationships between component scores for meat and alternatives (*r* = 0.113, *p* = 0.013), vegetables (*r* = 0.125, *p* = 0.006), whole grains (*r* = 0.330; *p* < 0.001) and fruit (*r* = 0.325; *p* < 0.001). Dietary intakes of all “core” food groups were significantly positively correlated in mother-child dyads, except for ‘dairy and alternatives’.

**Table 4:** Relationships between the quality of child’s diet and maternal habits in relation to diet quality scores and dietary intakes

Component scores	Correlation HEI-SG-C vs HEI-SG-M, total score (r-value)	Diet quality [ HEI-SG] adequacy components (/100)					Dietary Intake [serving/day] adequacy components (/100)					
		*Child’s cohort*	*Mother’s cohort*				*Child’s cohort*		*Mother’s cohort*			
		Mean (SD)	Mean	SD	r	*p*	Mean	SD	Mean	SD	r	p
Overall (total) score	0.176	64.3 (11.7)	57.48	(11.64)	0.176	< 0.001	–	–	–	–	–	–
Rice and alternatives	0.042	76.3 (23.6)	86.40	(15.53)	0.061	0.182	1.68	(0.72)	2.64	(0.68)	0.101	0.027
Whole grains	0.285	29.5 (37.3)	34.18	(33.01)	0.330	< 0.001	0.24	(0.37)	0.45	(0.56)	0.333	< 0.001
Meat and alternatives	-0.036	73.9 (30.8)	98.83	(5.10)	0.113	0.013	0.59	(0.36)	1.83	(0.58)	0.145	0.001
Dairy and alternatives	-0.88	70.9 (18.1)	63.57	(34.52)	0.028	0.541	1.43	(0.43)	0.25	(0.22)	0.047	0.301
Vegetables	0.084	75.3 (29.2)	62.84	(25.32)	0.125	0.006	0.72	(0.61)	0.77	(0.42)	0.158	< 0.001
Fruit	0.209	61.55(33.01)	63.47	(30.64)	0.325	< 0.001	0.55	(0.56)	0.85	(0.59)	0.336	< 0.001

*n* = 484 mother-child dyads

Table 5 shows that breastfeeding was the strongest predictor of diet quality, followed by household income, mother’s diet quality (HEI-SG-M) and maternal BMI. Breastfeeding has the strongest influence on the quality of a child’s diet, with a mean difference of 4.8 quality units between HEI-SG-C scores for children who had not been breastfed versus those that had. Vegetable intake (servings/d) in a child’s diet appeared to be associated with the child’s fruit intake- (servings/d), household income and maternal vegetable intake (servings/d).

**Table 5:** Multiple linear regression analyses, examining the potential predictors for a child’s diet quality and vegetable intake

Variables	Coeff	β	t	*p*	95% CI lower	95% CI upper	VIF
*Child’s diet quality*							
Breast feeding practice	4.778	0.195	4.587	< 0.001	2.731	6.826	1.055
Household income	2.311	0.157	3.68	< 0.001	1.077	3.545	1.062
HEI-SG-M (total) score	0.153	0.155	3.723	< 0.001	0.072	0.234	1.02
*Child’s vegetable intake (servings/d)*							
Child’s fruit intake (servings/d)	0.253	0.359	8.258	< 0.001	0.193	0.314	1.008
Household income	0.084	0.210	4.822	< 0.001	0.050	0.118	1.013
Mother’s vegetable intake (servings/d)	0.140	0.190	4.352	< 0.001	0.077	0.203	1.015

*n* = 484 pairs. Coeff = unstandardised coefficient; B = standard coefficient; t = t statistics; VIF =variance inflation factor; Dependent variable = child total diet quality score; R^2^ = 0.203 and for vegetable intake, R^2^ = 0.255. All multivariable regression models included: child’s sex, BMI and age were adjusted for covariates. For stepwise regression analysis, R^2^ = 0.066 for step 1; R^2^ = 0.122 for step 2; R^2^ = 0.156 for step 3; R^2^ = 0.181 for step 4; R^2^ = 0.193 for step 5; R^2^ = 0.203 for step 6

### Individual in-depth interviews (IIDIs)

The sociodemographic characteristics of mothers are shown in Table 1. Three-quarters of participants had full time jobs and needed their parents or domestic helper to daycare their children. Four key, consitent themes were identified, and the respective quotes are presented in Table 6 alongside representative quotes. These themes included “*Inculcating vegetable and fruit eating habits*”. Mothers claimed that liking for vegetables was inspired by their own mothers, highlighting that inclusion of vegetables and fruits in the diet from a young age such as toddlerhood period was pivotal to impart the vegetable eating habit (see Table 6). Mothers also shared the belief that if they ate vegetables together with their children would encourage them to eat vegetables (see Table 6). 

**Table 6:** Summary of the quotes related to shared themes from the individual in-depth interviews

1: Inculcating vegetable and fruit eating habits	• Because I have friends, particularly guy friends who don’t eat vegetables at all because they never have the habit to eat vegetables. Even though they say they can find supplements to replace this portion, but I still feel that, vegetables will still have certain kinds of minerals in it that is good for the body. [M282] • .To me, as long as you eat vegetables, I think it is good enough. As in all your meals throughout the day that you eat cannot be all carbo, or meat. [ M207] • Just trying a different type of vegetable. Because so far, the ones that we have tried I mean we need more. He’s growing you know. So, I want to introduce him to different varieties. [M454] • …. as long as my mother cooks vegetables, she will have portion of vegetable to eat. She will not reject also.”. [M207] • I follow what my mom taught me, and I believe that this is something how we grew up. So, she thinks that it is good enough. [M391] • There is another type of vegetable my mother-in-law says that is good for kids, hai taya. …. I think it is called the water cress, maybe the water cress vegetable. For the Chinese they think it is good for the children. [M430] • I guess, since young my mom has trained us well to eat a lot of vegetables rather than meat. Personally, I do enjoy the crunch of the greens.” [M454] • For dinner sometimes after dinner we will eat fruits because my daughter loves fruits. [M207] • For him for a healthy…like how I feel that I make a healthy diet for him. I ensure that his breakfast always has fruits. [M178] • She will eat apple and pear with us because every night we will have it so she will eat it as well. But on and off we will give her strawberries because she loves strawberries and some avocado. [M457] • Vegetables, let’s say for dinner my mother will cook 1 bed of vegetables and me and my mother and my daughter will just split. [M207] • if let’s say I am home then I would go to the supermarket to buy some vegetables and I will at cook at home… I will ensure that at least when he is with me at home, I will feed him at least a portion of starch, then a portion of vegetable and meat. [M282]
2: Perception about whole grains	• No preference because I am not a rice person. So, I will just eat whatever that is being cooked at home. [M454] • My mother-in-law thinks the brown rice is more suitable to aging people, and I eat what she prepares. [430] • I usually eat what my mother cooks, sometimes, she cooks brown rice, then I eat brown rice. [ M207] • I just followed my mom. Because, I don’t have any idea. So, she is the one that taught me how to cook for my baby. It is quite natural for me to just follow her. [ M282] • We do have brown rice, but we (referring to participant’s mother) don’t feed him that every time. He is too young right? The digestion system, you get to see the husk and all that, so we do in moderation. [M254] • Not really, because my mother also feels that those multigrain rice is a bit harder comparing to white rice. It is also because of my daughter who finds it hard. So maybe, it is not so easily digested by children. This is why we choose white rice over multigrain. [M207] • I was told that it is actually too young to introduce whole grain to a child because of the indigestion, it will harm him. [M391] • So not for young children because they need the energy. So, she (mother-in-law) does not want to feed him brown rice. [M430]
3: Breast is best-formula supports the rest	• I will try my best to express it out for her because that is the only thing that I can provide her, because most of the time I am at work and I don’t have really a lot of time to accompany her. That is the only thing that I could give it to her. (M207) • My husband asked me to stop. He sees me like very poor thing. Because I am alone at home, no one is helping me so, it is like, I need to pump and then I need to….so, my husband is saying, “Oh, you will be very busy, you need to prepare the food. So, maybe you slow down on the breastfeeding and then transit her to formula. [ M382] • I pumped up the breastmilk, then I put in the fridge, and then after the fridge I need to take out and warm it. Then she (mother-in-law) said there is a lot of wind inside the warmed-up milk that was stored in the fridge. So, after that she told me to feed him (son) on formula, because his stomach was better with formula milk. So, I just fully put him on formula. [M430] • I will express in the office. It is healthier. I think it is recommended. Most people say to feed for 6 months. [ M391] • For 6 months. Because I have a condition as well. I have hyperthyroidism. 6 months was my max that my doctor gave me to breastfeed her. [M457] • …and only at night before sleeping then we will use the formula plus the breast milk. To let her feel full so that she can drag her waking time later and later. [M346]
4: Aspiration against Pragmatism	• To me organic is…but of course I tried before…when I go for organic it is usually vegetables. [254] • I will let her eat all the healthy food, organic food. [M457] • Healthy food and healthy diet or balanced diet, no fried stuff or less fried stuff…. So, more vegetables, fruits as well as more organic stuff. [M391]. • For the snack, it used to be healthier, I gave him fruits. But I think nowadays because he tried the other things. He likes oat or muesli bars for kids. For that, I always bought the organic one. [M178] • Say example, maybe the organic because it is natural without any additives added to their growing so basically the nutrients of course are higher than those non-organic. [M346] • After all my experience, I feel that, I always buy organic one. Organic one then it will turn out perfect. [M382] • No. I mean, I know organic is better, but I don’t think that it is super important to eat organic. [M386] • Items that I buy. Organic rice and then if they have healthy snacks like organic rice snacks I would sometimes buy and then organic beans. [M396] • If I can afford organic food for her why not. I would think that, it is a healthier food than giving her other foods. [M457] • No. In Singapore it is very expensive to eat organic food. [M386] • Mostly it is for my son. But I will still buy and eat organic stuff in the basis that my son can take it. [M396]

The second theme was “*Perception about whole grains*”: Most mothers were not aware of the health benefits of whole grains despite much publicity has been given recently (see Table 6). ‘Brown rice’ would not be introduced because it was perceived to be harder to digest and unsuitable for their children, or even harmful to them by mothers and grandmothers. The timing to introduce whole grains was unclear. The third theme was “*Breast is best - formula supports the rest*”: The close mother-child bond associated with breastfeeding and the strong desire to give the best to their child was commonly quoted (see Table 6) by the interviewing mothers. Interviewing mothers were compelled to breastfeed their child for at least the first 6 months. A high level of social conformity to breastfeeding their child was observed in mothers. Mothers could feel lost if they quit breastfeeding before they were emotionally ready. Quitting breastfeeding created a sense of guilt in some mothers. The final theme was “*Aspiration against Pragmatism*” : Several mothers were strong supporters of organic foods, as they believed organic food were healthier, authentic, natural (free from pesticides and fertilizers) above the conventional form (see Table 6). Mothers aspired to purchase organic foods, however, ‘pricing’ could be a concern to some mothers. A pragmatic approach to deal with this, mothers would pay the higher price for the foods exclusively on their child, and not for the entire family members.

## Discussion

The overall findings of this mixed-method research suggested that ethnicity, lower maternal age, higher maternal BMI, lower household income, lower maternal education, and lack of previous breastfeeding were correlates for lower adherence of the child’s dietary habit to the national food-based guidelines. While maternal diet quality scores were statistically correlated with that of their children, this association was weak – perhaps underlining the range of other factors that appeared to associate with. Breastfeeding appeared to be the strongest predictor of a child’s diet quality, followed by household income, the mother’s diet quality, and maternal BMI. The current study is one among a range of limited studies examining the diet quality of mother-child pairs in Singapore. Thus, the findings are of wider applicability in the Singaporean context but may also present an opportunity for public health approaches in other developed countries. Importantly, the current work included parallel collection of qualitative data from mothers on their health beliefs which has helped uncover novel drivers and barriers for food provision within this population.

Previous work has suggested that Singaporean children of ethnic minorities have been shown to display less ideal dietary patterns than those of Chinese ethnic origin [15, 46–49]. The GUSTO Study reported that younger mothers in the age range (18–29 years old) tended to offer more ‘Easy-to-prepare foods’ to their children, possibly owing to limited knowledge and experience with infant nutrition [48].

Lower maternal education attainment and household income were unfavourable to the diet quality of their offspring [15]. Breastfed children exhibited a better overall diet, and conversely, children of mothers with higher pre-pregnancy BMI had poorer diets [15]. Our results echo the previous findings reported in GUSTO [50] and are generally in line with previous observations from the UK [7, 51], Japan [52], and Australia [53] where a child’s diet pattern was associated with maternal characteristics and a diet of high adherence to the national food guidelines were characterised by more consumption of fruit, vegetables, and less discretionary foods. More recent nutritional screening studies in Southeast Asia have also highlighted major limitations in dietary habit in infants, including those of complementary feeding age [54–56].

A lower diet quality of Malay descendants has also been reported in children of school age [46] and that seems to be related to cultural food preferences [15]. However, the absolute number of mother-child dyads from minority ethnic backgrounds were low in the current study (Indians (*n* = 19) and Malays (*n* = 32)), which may have limited the potential to detect meaningful differences between groups.

In line with previous studies, we found evidence that younger maternal age is associated with more unhealthy diet patterns [7, 48, 49, 53]. Mothers of the younger age group (i.e., 18–29 years old) in this cohort tended to have lower education and fell within a lower-income household category. These additional sociodemographic factors have shown to negatively influence the diet patterns in infants and young children [8, 51]. Maternal education has been recognised as a strong predictor of infant feeding practices and the quality of children’s diets [57]. In this cohort, children of highly educated mothers had statistically higher median total diet quality scores. After adjusting for biological and socio-demographic factors, the “effect” of maternal education did not appear to be statistically significant. Mothers with higher educational attainment could be more aware of recommendation for complementary diets, or possibly more able to act on such guidelines [15, 17]. There was a limited range of maternal education levels seen in this cohort, with two-thirds of mothers being degree holders. While these findings should therefore be interpreted cautiously, a wide range of previous work has also suggested associations of maternal education with infant diet quality in instances where a wider range of education levels exist [58–61]. Additional studies have also shown that children with poorer diets were more commonly found in families with higher degrees of financial difficulty and mothers of lower education [17, 51–53]. Unhealthy dietary patterns composed of highly processed foods, fast foods, and sugary beverages observed in socially deprived young children are related to maternal poor nutrition knowledge, which may drive practice unhealthy food provisioning practice [62]. Regardless, household income in this cohort was an important correlate for a child’s diet quality and identified as the 2nd significant predictor. The present work confirms that the association between household income and the diet quality of the children is also apparent in infants and toddlers.

Consistent with previously reported study [57], breastfeeding practice seems to be a significant correlate for a child’s diet quality in this cohort, and it was the strongest predictor of a child’s diet quality. Breastfeeding practice is positively associated with a child’s vegetable and fruit intake [63–66]. Children in this cohort who had been breastfed exhibited a better diet quality, contributing by fruit, dairy and alternatives, lower sodium, saturated fat, and total fat. These findings are generally in line with previous observations that mothers who practise breastfeeding for a longer duration are more likely to adhere to national dietary guidelines and tend to follow a more prudent diet behaviour, such as consuming more vegetables or a greater variety of fruit and vegetables [63, 66]. Prolonged breastfeeding has been linked with a better-quality diet in children, characterised by higher vegetable and fruit intake in later life [65]. There is little empirical evidence on how a child’s diet changes with prolonged breastfeeding, and this may be an important area for further research. Previous work has suggested a potential conflict between extended breastfeeding delaying the start of complementary feeding, which could affect both dietary transition, as well as early life exposure to foods that might shape lifelong health trajectories for the infant [67–69].

Of note, individual in-depth interviews (IIDIs) confirm that breastfeeding provided mothers with an avenue to build stronger mother-child bonding, a way for them to express their continual involvement in nutritional care and, to some extent, for some mothers to take better control over the well-being of their children. Mothers taking part in IIDIs mostly practiced breastfeeding beyond 6 months, believed that vegetables and fruit are important components in a healthy diet, and that weaning was an opportune time to promote the acceptance of these foods [70].

Mothers in the IIDIs expressed a strong desire to nurture healthy diet habits by setting a role model, however, working conditions, family circumstances, and time constraints were commonly cited as barriers to making the lifestyle changes to improve their diets. In this study, a positive association was also observed between the habit of purchasing organic food and a child’s diet quality, mainly contributed by the higher component scores for vegetables and whole grains. Studies have shown that organic food supporters are health-conscious, sceptical about preservatives and chemical residues, and more receptive to making diet changes to improve health [71, 72]. Mothers with young children are willing to pay higher prices for organic produce when they perceive organic foods are healthier and natural [73].

Maternal diet quality was noted to be a significant correlate for the diet quality of a child and was also related to whole grain and fruit component scores. These dietary factors are key targets within national food-based dietary guidelines [41], with low habitual intake noted to relate to higher global risk of mortality and healthy life years lost. Taste inferiority is often identified as the key barrier to whole grain consumption in adult studies [74, 75]. Mothers were unfamiliar with wholegrain products and suggested that they found it hard to identify and/or to incorporate them into their diets. Further IIDI insights suggested that mothers and grandmothers (as secondary caregivers) frequently believed that whole grains were not suitable for infants and toddlers because of their coarse texture (particularly brown rice) and perceived indigestibility. The organoleptic properties of whole grains have been proposed as the main reason for them being rejected by mothers [76]. Studies examining the benefits of whole grains in infants and toddlers are limited, with some evidence of improved glycaemic control [77], and management of functional constipation [78]. Whole grains contribute to a higher intake of several nutrients and have previously been suggested to improve infant diet quality [79].

For fruit intake, approximately a quarter of mother-child dyads met the daily recommendation for servings of fruit, compared to 1 in 10 for whole grains recommendations [25]. Consistent with findings previously reported, women who adhered to healthy diets with higher intakes of fruit and vegetables were more likely to have children who had comparable diets [7, 80]. Our results suggested a link between mothers’ vegetable intake was an independent predictor for their child’s vegetable intake. However, children were three times more likely to meet vegetable guidelines as compared to their mothers within the current cohort (45% and 15% met guidelines respectively) [25]. Mothers in this cohort were keen to inculcate vegetable-eating habits though they were not necessarily compliant themselves. This observation could exemplify a maternal focus on provision of prudent dietary choices for the infant without the same level of concern for their own dietary habits. This would appear a rational approach to target improvement of both maternal and child dietary habit in future public health interventions, due to the potential for both positive food choices to be available in-home, as well as maternal motivation for prudent eating to be high [81, 82].

### Strengths and limitations

This study has a large sample size and includes a purpose-built healthy eating index to assess adherence to national food and nutrient guidelines in Singaporean children in the age range of 6 to 24 months. This work has added to recent knowledge on maternal feeding practices in complementary feeding in highly-developed countries. A major strength of this study is its mixed-method design in which both quantitative and qualitative techniques were used in data collection to gather a more comprehensive insight into the relationship of diet quality in mother-child dyads. Such an exploratory approach could potentially support development of future, validated and targeted data collection tools to expand on this work [83]. Measures to enhance the relevance and appropriateness of diet quality scoring approaches included: (i) relatively large sample size with a range of economic diversity; (ii) dietary intake assessment using validated FFQs; (iii) scoring calculations considering the physical activity, pregnancy with energy-adjustment in a national food/ nutrient-based HEI construct to mitigates issues related to dietary data collection methodology; (iv) onsite anthropometric measurements carried by the trained research coordinator (ALFC). The authors also note several limitations within the current work. Perhaps most importantly, the cross-sectional design of the study precludes evaluation of current habits on longer-term health outcomes. It also means that the impact of earlier life exposures (e.g. breastfeeding) cannot be easily disaggregated from current health status or dietary behaviours in infants. The use of FFQs is prone to over/underreporting of foods due to social desirability, or conformation of the peers’ norms is not uncommon, thus the potential for reporting biases is always possible. We have attempted to limit this potential through our approach to adjustment of diet quality score component by energy intake, an approach that has formed the basis of the well-established HEI assessment in the United States [84].

As noted above, limited sub-group size reduces the certainty of statistical analyses carried out, particularly in the case of ethnic backgrounds and education level. Participants on the current study came from one predominant ethnic background (Chinese) and tended to be of higher socio-economic status. As such, our findings should be conservatively considered in relation to wider populations. Future studies should aim to recruit a larger number of ethnic minority groups resident in Singapore and include more participants across the full range of educational backgrounds. Since grandparental support in day caring of their grandchildren is evident in Singapore [85], further studies should also include collection of qualitative information grandparents and other major caregivers.

## Conclusions

Maternal and infant diet quality are intricately linked to each other. Factors such as maternal age, education, BMI status, and household income appear to affect the quality of the child’s diet. Healthy feeding practices and healthy dietary trajectories should be promoted during complementary feeding, particularly among socially disadvantaged populations.

## Supplementary Information

Below is the link to the electronic supplementary material.

Supplementary Material 1

## Data Availability

Data cannot be shared openly but are available on request from authors.

## Declarations

### Employment

ALFC and AL are employees of Nestlé Singapore.
