# Supplementary material for: A mixed-method approach to assess factors associated with food provision to children during weaning in Singapore
Source: Eur J Nutr. 2026 Mar 2;65(2):76. doi: 10.1007/s00394-026-03927-7 (PMC12953361; doi:10.1007/s00394-026-03927-7)
Supplement: Supplementary file 1 — Supplementary Material 1 [file 394_2026_3927_MOESM1_ESM.docx]

Supplementary Table A: Evaluation of Child Healthy Eating Index (HEI-SG-C) Score by the Maternal Education Attainment (n=484)

| Component | Basic ^1^  (n=80) | Intermediate^2^ (n=80) | Advance I^3^  (n=267) | AdvanceII^4^  (n=57) |  |
| --- | --- | --- | --- | --- | --- |
|  | Median | Median | Median | Median |  |
|  | IQR | IQR | IQR | IQR | p value |
| Rice & Alt | 78.7 | 77.9 | 82.7 | 75.9 | 0.430 |
|  | (58.7-100) | (57.9-100) | (63.1-100) | (54.8-100) |  |
| Whole grains | 0.0 | 7.2 | 7.7 | 17.2 | 0.155 |
|  | (0-32.7) | (0-63.4) | (0-55.8) | (0-53.2) |  |
| Meat & Alt | 89.0 | 76.5 | 91.2 | 93.0 | 0.130 |
|  | (60.9-100) | (37.6-100) | (56.7-100) | (40.8-100) |  |
| Dairy & Alt | 70.9 | 72.1 | 71.2 | 68.3 | 0.412 |
|  | (58.5-82) | (60.2-84.1) | (58.9-84.9) | (53.9-78.3) |  |
| Vegetables | 74.1 | 90.4 | 88.2 | 100.0 | 0.053 |
|  | (41.2-100) | (47.4-100) | (56.0-100) | (69.3-100) |  |
| Fruit | 58.5^a^ | 51.8^a^ | 60.5^a^ | 93.5^b^ | <0.001 |
|  | (21.2-100) | (28-96.4) | (35.7-96.8) | (62.3-100) |  |
| Sodium | 100 | 100 | 100 | 100 | 0.036 |
|  | (0-100) | (0-100) | (100-100) | (100-100) |  |
| Total Fat | 100.0 | 100.0 | 100.0 | 100.0 | 0.949 |
|  | (0-100) | (20.8-100) | (0-100) | (0-100) |  |
| Sat. Fat | 0.0 | 0.0 | 0.0 | 0.0 | 0.651 |
|  | (0-47.6) | (0-41.5) | (0-32.7) | (0-50.0) |  |
| Added Sugar | 94.3^a^ | 96.7^ab^ | 97.6^b^ | 98.2 ^b^ | <0.001 |
|  | (85.4-97.9) | (91.1-98.9) | (93.9-99.3) | (93.3-99.7) |  |
| Overall (Total)* | 61.2^a^ | 62.7 ^ab^ | 65.3^b^ | 66.8^b^ | 0.009 |
|  | (58.5-63.9) | (59.9-65.4) | (64.0-66.6) | (63.4-70.1) |  |

| *ANOVA test, with mean and 95% confidence interval. Alt refers alternative. Sat. refers to saturated. |
| --- |

^1^Basic education refers to mothers who were schooler leavers. ^2^ Intermediate education refers to mothers who were polytechnic or diploma holders. ^3^ Advance I education refers to mothers who were degree holders. ^4^ Advance II education refers to mothers who were postgraduate degree holders.

Sub-groups with different superscripts (a/b) for education attainment in a single row are statistically different (p < 0.05) by Kruskal-Wallis tests with Bonferroni’s post hoc test.

Supplementary Table B: Evaluation of Child Healthy Eating Index (HEI-SG-C) Scores by the Household Income (n=482)

| Component | Level I ^1^  (n=29) | Level II^2^  (n=217) | Level III ^3^  (n=183) | Level IV ^4^  (n=53) |  |  |
| --- | --- | --- | --- | --- | --- | --- |
|  | Median | Median | Median | Median |  |  |
|  | IQR | IQR | IQR | IQR | p value |  |
| Rice & Alt | 83.5 | 81.4 | 80.9 | 74.3 | 0.420 |  |
|  | (65.1-100) | (61.7-100) | (60.8-100) | (52.3-97.4) |  |  |
| Whole grains | 11.2 | 2.0 | 12.8 | 22.2 | 0.053 |  |
|  | (0-31.6) | (0-47.6) | (0-83.8) | (0-53.2) |  |  |
| Meat & Alt | 74.3 | 88.5 | 90.9 | 89.3 | 0.600 |  |
|  | (57.3-100) | (47.2-100) | (55.9-100) | (59.8-100) |  |  |
| Dairy & Alt | 66.8 | 71.8 | 69.8 | 71.2 | 0.453 |  |
|  | (55-78.0) | (58.6-86.9) | (59-81.8) | (62.1-79.6) |  |  |
| Vegetables | 58.7^a^ | 80.1^ab^ | 99.7^b^ | 100.0^b^ | 0.009 |  |
|  | (26.2-100) | (51.9-100) | (59.1-100) | (63.6-100) |  |  |
| Fruit | 61.2 | 62.4 | 60.9 | 78.6 | 0.274 |  |
|  | (29.4-100) | (26.3-100) | (38.2-100) | (37.0-100) |  |  |
| Sodium | 0^a^ | 100^b^ | 100^b^ | 100^b^ | 0.003 |  |
|  | (0-100) | (100-100) | (100-100) | (100-100) |  |  |
| Total Fat | 100.0 | 100.0 | 100.0 | 100.0 | 0.199 |  |
|  | (41.2-100) | (0-100) | (25.5-100) | (32.1-100) |  |  |
| Sat. Fat | 0.0 | 0.0 | 0.0 | 0.0 | 0.422 |  |
|  | (0-5.7) | (0-44.7) | (0-32.7) | (0-39.4) |  |  |
| Added Sugar | 89.9^a^ | 96.5^b^ | 98.0^b^ | 97.8^b^ | <0.001 |  |
|  | (83.2-97.0) | (92.9-98.9) | (94.2-99.3) | (93.4-99.6) |  |  |
| Overall (Total)* | 57.7^c d^ | 62.9^c^ | 66.6^a b^ | 66.4^a^ | <0.001 |  |
|  | (53.7-61.8) | (61.2-64.6) | (65.1-68.1) | (63.1-69.8) |  |  |
| Two mothers did not disclose their household income. *ANOVA test, with mean and 95% confidence interval; Alt refers to alternative. Sat. refers to saturated. | | | | | | |

^1^ Level I refers to household income <$ 3500. ^2^ Level II refers to household income $3501- ≤ $9000. ^3^ Level III refers to household income $9001- ≤ $15000. ^4^ Level IV refers to household income $15001- ≥ $20001

Sub-groups with different superscripts (a/b) (c/d) for income in a single row are statistically different (p < 0.05) by Kruskal-Wallis tests with Bonferroni’s post hoc test

Supplementary Table C: Evaluation of Child Healthy Eating Index (HEI-SG-C) Score by the Breastfeeding Practice of the Mothers (n=484)

| Component | First 6 months (n=336) | Ever Breastfeeding (n=136) | Never  (n=12) |  |
| --- | --- | --- | --- | --- |
|  | Median | Median | Median |  |
|  | IQR | IQR | IQR | p value |
| Rice & Alt | 79.7 | 84.1 | 93.1 | 0.235 |
|  | (59.5-100) | (62.5-100) | (82.3-100) |  |
| Whole grains | 8.5 | 4.2 | 3.1 | 0.660 |
|  | (0-53.2) | (0-47.8) | (0-76.1) |  |
| Meat & Alt | 89.7 | 88.8 | 71.5 | 0.576 |
|  | (51.2-100) | (56.7-100) | (57.6-89.7) |  |
| Dairy & Alt | 72.7^a^ | 68.3^b^ | 62.6^ab^ | 0.004 |
|  | (60.1-86.8) | (55.1-77.0) | (58.8-72.9) |  |
| Vegetables | 89.6 | 95.8 | 65.5 | 0.304 |
|  | (53.7-100) | (51.6-100) | (47.7-85.9) |  |
| Fruit | 68.7 | 56.1 | 43.0 | 0.051 |
|  | (35.4-100) | (29.1-90.8) | (26.2-78.1) |  |
| Sodium | 100^a^ | 100^b^ | 100^ab^ | 0.008 |
|  | (100-100) | (0-100) | (0-100) |  |
| Total Fat | 100.0 | 100.0 | 0.0 | 0.030 |
|  | (20.8-100) | (0-100) | (0-100) |  |
| Sat. Fat | 4.5^a^ | 0.0^b^ | 0.0^b^ | <0.001 |
|  | (0-52.4) | (0-0) | (0-0) |  |
| Added Sugar | 97.3 | 97.1 | 97.3 | 0.475 |
|  | (93.2-99.3) | (91.4-98.8) | (95.2-99.2) |  |
| Overall (Total)* | 66.1^a^ | 60.6^b^ | 57.5^b^ | <0.001 |
|  | (64.9-67.4) | (58.8-62.4) | (50.3-64.7) |  |

*ANOVA test, with mean and 95% confidence interval; Alt refers alternative; Sat. refers to saturated.

Sub-groups with different superscripts (a/b) for breastfeeding practice in a single row are statistically different (p < 0.05) by Kruskal-Wallis tests with Bonferroni’s post hoc test.

First 6 months refers to the children who were breastfed for the first 6months. Ever breastfeeding refers to children who were breastfed for less than 6 months. Never refers to children who were never breastfed.
